# Elucidating the critical role of gut microbiota in the pathogenesis of bacterial pneumonia: insights from a Mendelian randomization analysis mediated by immune cell

**DOI:** 10.1186/s12879-025-10758-0

**Published:** 2025-03-18

**Authors:** Xin Gao, Changle Wang, Bingxin Pan, Yawen Liu, Shuo Yuan, Shaoru Zheng, Dongmei Yu, Lujuan Han, Zhaohua Meng

**Affiliations:** 1 The Second Department of Infection, The Second Hospital of Hebei Medical University, Heping West Road 215, Shijiazhuang, 050061 China; 2 Department of Pathogenic Biology, Hebei Medical University, Zhongshan Road 361, Shijiazhuang, 050017 China; 3 Public Research Platform, School of Basic Medicine Sciences, Hebei Medical University, Zhongshan Road 361, Shijiazhuang, 050017 China; 4 Department of Clinical Laboratory, Hui’an County Hospital, Huixing Street 582, Quanzhou, 362100 China; 5 215# Heping West Road, Shijiazhuang, China

**Keywords:** Gut microbiota, Immune cells, Bacterial pneumonia, Mendelian randomization

## Abstract

**Background:**

The gut microbiota (GM) is recognized as a critical factor in sustaining overall health and regulating the immune system. However, the precise function of GM in the pathogenesis of bacterial pneumonia (BP), as well as the potential involvement of immune cells in these mechanisms, remains inadequately understood. Given that BP represents a substantial public health issue, elucidating the protective role of the gut microbiota against this condition is of considerable significance.

**Methods:**

We employed a bidirectional two-sample univariate Mendelian randomization (UVMR) approach to investigate the potential causal relationship between GM and BP. Furthermore, we integrated UVMR with multivariate Mendelian randomization (MVMR) analysis to assess the mediating role of immune cells in the pathway linking GM to BP risk. We additionally performed a reverse analysis to exclude GM that could exhibit a reverse causal relationship with BP.

**Results:**

Mendelian randomization (MR) analysis identified 18 GM significantly associated with BP, with 8 of these bacterial taxa linked to a reduced risk and 10 associated with an increased risk. Additionally, 50 immune cell traits exhibited suggestive associations with BP, with 27 immune cells potentially conferring protection and 23 immune cells potentially augmenting risk. Importantly, mediation MR analysis revealed that the protective effect of *Clostridia* on BP was predominantly mediated by the proportion of HLA DR + Natural Killer cells within CD3- lymphocytes (HLA DR + Natural Killer %CD3- lymphocytes) (Total effect IVW: OR = 0.724, 95% CI [0.552, 0.950], *P* = 0.020). The evaluation of the mediation effect revealed an effect size of -0.025 (95% CI [-0.061, -0.000]), with a mediation effect ratio of 7.143%.

**Conclusion:**

The study identified specific components of the GM that confer a protective effect against BP. It revealed that the subsets of HLA DR + Natural Killer %CD3- lymphocytes are modulated by *Clostridia*, thereby enhancing the host’s immune defense against BP.

**Supplementary Information:**

The online version contains supplementary material available at 10.1186/s12879-025-10758-0.

## Introduction

Bacterial pneumonia (BP), characterized by lung infection caused by pathogens such as *Streptococcus pneumoniae*, *Haemophilus influenzae*, and *Staphylococcus aureus*, is associated with significant morbidity and mortality, particularly in vulnerable populations such as children, the elderly, and immunocompromised individuals [1, 2]. The clinical manifestations of BP encompass fever, cough potentially accompanied by yellow-green sputum, chest pain, dyspnea, fatigue, chills, and diaphoresis. Additionally, some patients may present with gastrointestinal symptoms such as nausea, vomiting, and diarrhea [3, 4]. Currently, the primary therapeutic approaches for BP include antibiotic treatment, supportive care, and adjunctive therapies [5–7]. However, these methods exhibit several limitations, including the emergence of antibiotic resistance [8] and the potential for long-term antibiotic use to induce dysbiosis of gut microbiota (GM), allergic reactions, and other adverse effects [9]. Consequently, it is imperative to comprehend the factors influencing the incidence and mortality rates of BP to inform effective management and intervention strategies. Furthermore, there is an urgent need to develop more effective and safer treatment programs to alleviate patient suffering and enhance their quality of life.

Given these therapeutic challenges, emerging research has increasingly focused on the immunomodulatory potential of GM as a novel intervention avenue [10–13]. The GM interacts dynamically with host immunity to modulate systemic health and disease pathogenesis [14, 15]. Key mechanisms involve short-chain fatty acids production and direct regulation of immune cell development and function [16, 17]. During pulmonary infections, GM-activated immune cells (B cells, T cells) migrate to lung tissue via circulatory pathways, initiating localized immune responses [18, 19]. This gut-lung axis maintains a critical balance between Treg-mediated anti-inflammatory protection and Th17-driven pathogen clearance. Chronic dysregulation of this axis promotes airway inflammation and tissue remodeling characteristic of chronic obstructive pulmonary disease and asthma [20–22]. These findings collectively demonstrate bidirectional regulation of host immunity via the gut-lung axis, with immune cells orchestrating this cross-tissue communication as cellular mediators [23, 24].

According to these findings, we posit the hypothesis that there exists a potential causal link between GM, immune cells, and BP. Our objective is to conduct an in-depth investigation of these interconnections and to identify potential targets within the GM and immune system for early detection and therapeutic intervention in clinical settings.

Mendelian randomization (MR) serves as a robust method for causal inference, utilizing genetic variation as instrumental variables (IVs) to examine potential causal relationships between exposure factors and outcomes [25]. In this study, GM and immune cells were chosen as exposure variables, while BP was selected as the outcome variable for MR analysis. We utilized MR to systematically investigate the potential mediation effect of GM on the risk of developing BP through the modulation of immune cells. These findings provide novel insights and potential therapeutic targets for the prevention and management of BP.

## Methods

### Study design

This study utilized a two-phase MR design to investigate the potential relationship between GM and genetic susceptibility to BP, as well as the mediating effects of immune cell traits. The first phase of the study employed a bidirectional two-sample univariate Mendelian randomization (UVMR) approach, adhering to the fundamental principles of MR analysis, to assess the causal impact of GM on BP. The selection of single nucleotide polymorphisms (SNPs) was conducted with careful consideration of specific criteria, including strong correlation with GM, indirect influence on BP through GM, and lack of association with potential confounding variables. Additionally, a backward analysis was carried out to eliminate GM factors that may have a reverse causal relationship with BP. In the subsequent phase, UVMR was utilized in conjunction with multivariate Mendelian randomization (MVMR) analysis to evaluate the intermediary role of immune cells in the relationship between GM and BP risk, quantifying the magnitude of influence of each mediator and its proportion. This investigation adhered closely to the STROBE-MR guidelines: Strengthening the Reporting of MR in Observational Studies in Epidemiology [26] (Supplementary file 1: Table S1).

### Data sources

The study utilized data obtained from a comprehensive population health survey conducted in Finland, which employed a large, uniform population cohort to correlate human genotypes with fecal metagenomic data. Through the application of a genome-wide association study (GWAS), correlations between human genotypes and gut microbial abundance were identified in a sample size of 5,959 individuals (FINRISK 2002, FRO2). The study protocol of FR02 was approved by the Coordinating Ethical Committee of the Helsinki and Uusimaa Hospital District (Ref. 558/E3/2001). All participants signed an informed consent. The study was conducted according to the World Medical Association’s Declaration of Helsinki on ethical principles. The study utilized a genome-wide significance threshold of *P* < 5.0 × 10^− 8^ and identified 471 distinct GTDB taxa spanning 11 phyla, 19 classes, 24 orders, 62 families, 146 genera, and 209 species (GCST90032172 to GCST90032644) [27]. BP data were sourced from finn-b-J10_PNEUMOBACTEROTH (https://gwas.mrcieu.ac.uk/datasets/finn-b-J10_PNEUMOBACTEROTH/; *n* = 196,382), comprising 7,514 BP cases and 188,868 control cases [28]. Data on immune cell characterization were collected from the GWAS catalog for 731 immune cell traits, which were classified into six distinct groups: B cells, cytotoxic dendritic cells (CDCs), T cells in the maturation phase, monocytes, myeloid cells, and TBNKs (comprising Natural Killer cells, T cells, and regulatory T cells). These traits encompassed 118 absolute cell counts (AC), 192 relative cell counts (RC), 389 median fluorescence intensities (MFI) of surface antigen levels, and 32 morphologic parameters (MP) [29]. The participants in this study were all European descent.

### Genetic instrumental variable selection

Exposed and mediated SNPs were identified as IVs with a significance threshold of *P* < 1.0 × 10^− 5^ [30]. The independence of the selected IVs was ensured by utilizing the “TwoSampleMR” package (version 0.6.1) in R and setting the threshold for linkage disequilibrium at R2 < 0.001 and the clustering distance at 10,000 kb. Subsequently, the F-statistic was calculated using the formula F = R2 (N-k-1) / [ (1-R2) k] to evaluate the strength of the selected SNPs. In the current model, R2 denotes the fraction of variance elucidated by individual SNPs, N signifies the sample size of the GWAS, and k represents the count of SNPs. SNPs with F-values exceeding 10 were exclusively retained [31]. Conversely, in the subsequent analysis, a more rigorous screening threshold was applied, selecting SNP loci with a *P* < 5.0 × 10^− 6^ as IVs, while maintaining consistency with the screening criteria employed in the initial analysis.

### UVMR and MVMR analysis

The inverse variance weighting (IVW) method was utilized in this study as the primary analytical approach to investigate the causal association between GM and BP. Additionally, four other methods, namely the weighted median method, the weighted mode method, the MR-Egger regression method [32], and the simple mode method [33] were employed as supplementary analytical tools. Binary outcomes were expressed as odds ratios (OR) with corresponding 95% confidence intervals (CI), while continuous outcomes were reported as β-values. In the MVMR analysis, the utilized primary analytical approach was multivariate inverse variance weighting (MV-IVW). Statistical significance was defined as the *P*-value < 0.05, indicating a potential causal association.

### Mediation MR analysis

A two-step MR analysis was employed to investigate the indirect impact of GM on BP via immune cells. The initial step involved estimating the influence of GM on immune cells (β1). In the subsequent phase of the study, an additional analysis was conducted to assess the impact of immune cells on BP (β2). By integrating the established causal relationship between GM and BP (β) as determined through the UVMR technique, we computed the mediating influence of immune cells in the GM-BP pathway, along with their relative contribution, denoted as [β1 × β2] / β, employing the “product of coefficients” methodology [34]. The standard error of the mediating effect was derived through the application of the delta method [35].

### MR sensitivity analysis

Sensitivity analyses were conducted utilizing MR-Egger regression, the leave-one-out, and the MR-PRESSO methods. Cochran’s Q statistic was computed for each SNP to evaluate heterogeneity, while the *P*-value of the intercept test in MR-Egger regression was employed to assess horizontal pleiotropy. The MR-PRESSO method was employed to address potential horizontal pleiotropy in the chosen IVs [36]. A significance level of *P* < 0.05 for heterogeneity or pleiotropy serves as an indicator. The presence of pleiotropy could undermine the establishment of causality. The analyses were conducted utilizing the “TwoSampleMR”, “MR-PRESSO”, and “MendelianRandomization” packages within the R software environment (version 4.3.2) [36].

## Results

### Causal associations of GM with BP

**Fig. 1:**
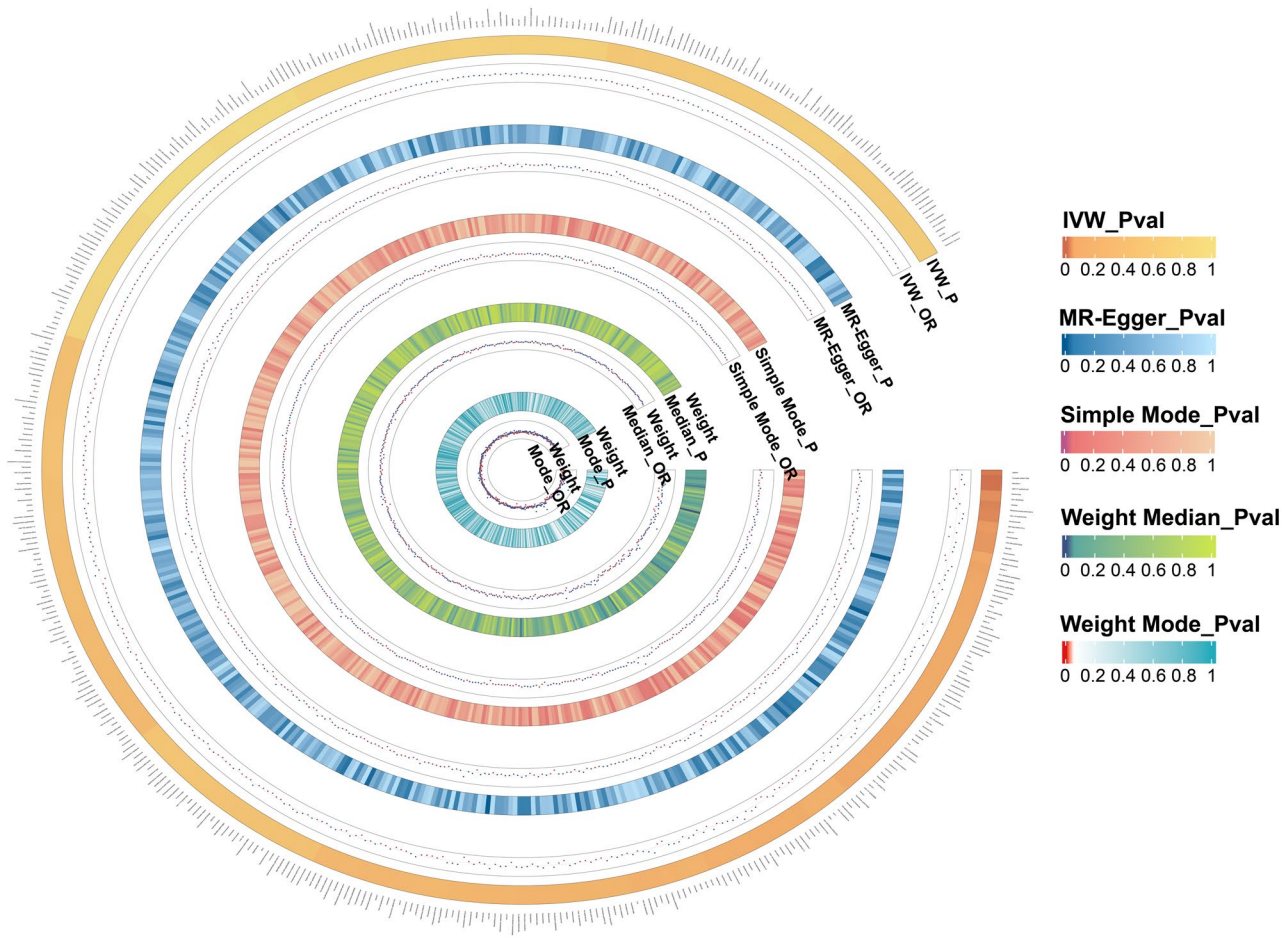
All results of gut microbiota. Our study is based on five analysis methods. Therefore, this figure displays the *P*-values and OR value of these methods. The five concentric heatmaps from outer to inner represent the IVW, the MR-Egger, the Simple Mode, the Weight Median and the Weight Mode analysis results of the gut microbiota, respectively. The darker the color, the more significant the results. OR, odds ratio; MR, Mendelian randomization; IVW, inverse-variance weighted

**Fig. 2:**
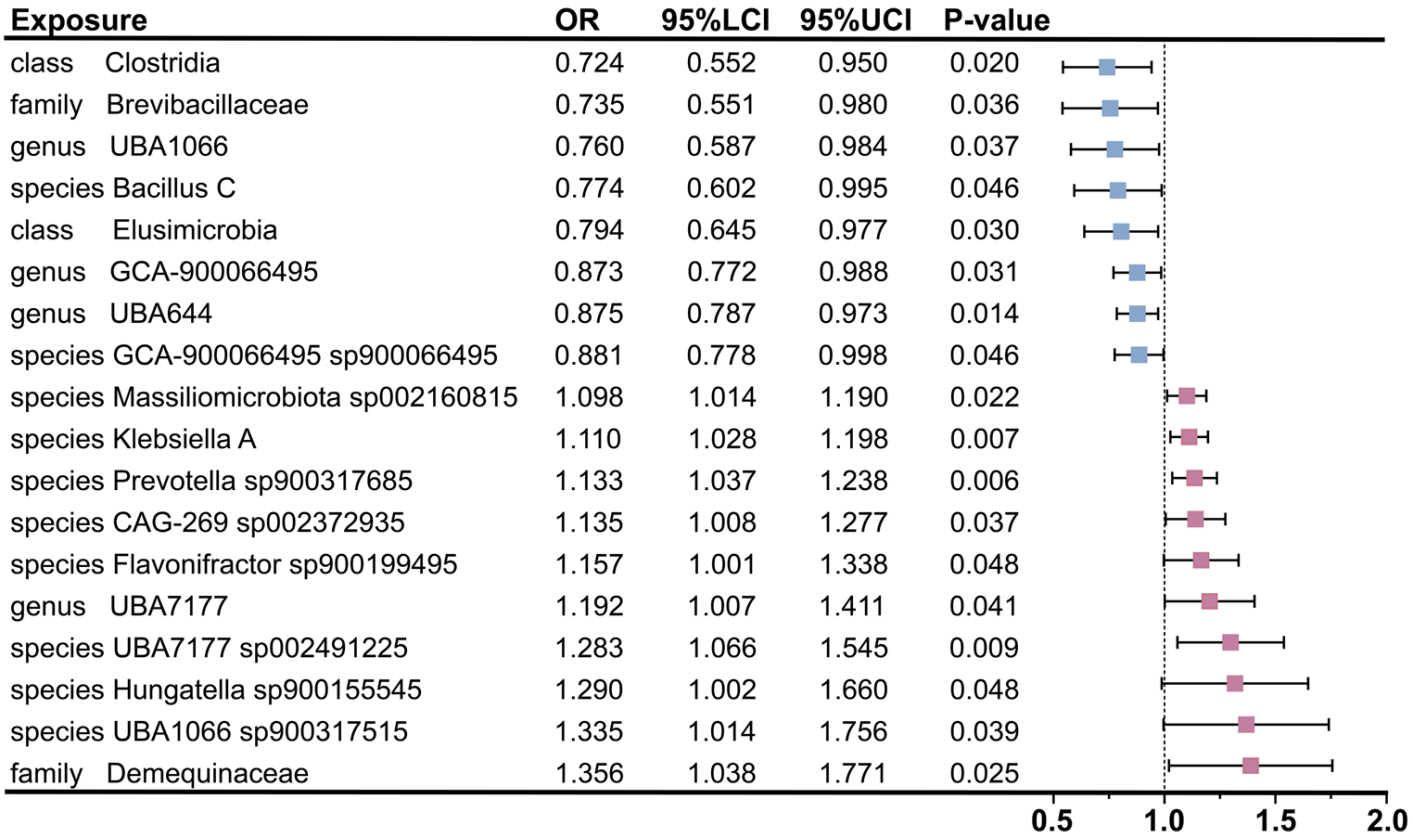
Mendelian randomization analyses show causal effects between gut microbiota and bacterial pneumonia. The little squares colored in pink and blue indicate positive and negative ORs respectively from the IVW analysis (truncated at *P*-value < 0.05). OR, odds ratio; IVW, inverse-variance weighted; LCI, lower confidence intervals; UCI, upper confidence intervals

In this study, 9,078, 17,565, and 4,257 SNPs were selected as study variables to investigate the interactions among 471 species of GM, 731 immune cells, and BP (Supplementary file 1: Table S2-4). As illustrated in Fig. 1, we performed a comprehensive analysis of the 471 GM species. Following the identification of 18 GM species significantly associated with BP, we primarily employed the IVW method to assess the causal effect of GM on BP. These findings indicated that the presence of the class *Clostridia* (OR = 0.724; 95% CI [0.552, 0.950]; *P* = 0.020), the family *Brevibacillaceae* (OR = 0.735; 95% CI [0.551, 0.980]; *P* = 0.036), the genus *UBA1066* (OR = 0.760; 95% CI [0.587, 0.984]; *P* = 0.037), the species *Bacillus C* (OR = 0.774; 95% CI [0.602, 0.995]; *P* = 0.046), the class *Elusimicrobia* (OR = 0.794; 95% CI [0.645, 0.977]; *P* = 0.030), the genus *GCA-900,066,495* (OR = 0.873; 95% CI [0.772, 0.988]; *P* = 0.031), the genus *UBA644* (OR = 0.875; 95% CI [0.787, 0.973]; *P* = 0.014), and the species *GCA-900,066,495* sp*900066495* (OR = 0.881; 95% CI [0.778, 0.998]; *P* = 0.046) was significantly associated with a reduced risk of BP. Simultaneously, the following bacterial species were found to be positively correlated with an increased risk of BP: the species *Massiliomicrobiota* sp*002160815* (OR = 1.098; 95% CI [1.014, 1.190]; *P* = 0.022), the species *Klebsiella A* (OR = 1.110; 95% CI [1.028, 1.198]; *P* = 0.007), the species *Prevotella* sp*900317685* (OR = 1.133; 95% CI [1.037, 1.238]; *P* = 0.006), the species *CAG-269* sp*002372935* (OR = 1.135; 95% CI [1.008, 1.277]; *P* = 0.037), the species *Flavonifractor* sp*900199495* (OR = 1.157; 95% CI [1.001, 1.338]; *P* = 0.048), the genus *UBA7177* (OR = 1.192; 95% CI [1.007, 1.411]; *P* = 0.041), the species *UBA7177* sp*002491225* (OR = 1.283; 95% CI [1.066, 1.545]; *P* = 0.009), the species *Hungatella* sp*900155545* (OR = 1.290; 95% CI [1.002, 1.660]; *P* = 0.048), the species *UBA1066* sp*900317515* (OR = 1.335; 95% CI [1.014, 1.756]; *P* = 0.039), and the family *Demequinaceae* (OR = 1.356; 95% CI [1.038, 1.771]; *P* = 0.025) (Fig. 2, Supplementary file 1: Table S5). Additionally, leave-one-out analyses demonstrated that no individual SNP significantly influenced the overall effect of GM on BP (Supplementary file 2: Fig. S1). In the reverse MR analysis, we found insufficient evidence to support a causal effect of BP on the identified 18 GM in the forward MR analysis. Detailed results of the reverse MR analysis were presented in Supplementary file 1: Table S6.

### Causal effects of immune cells on BP

**Fig. 3:**
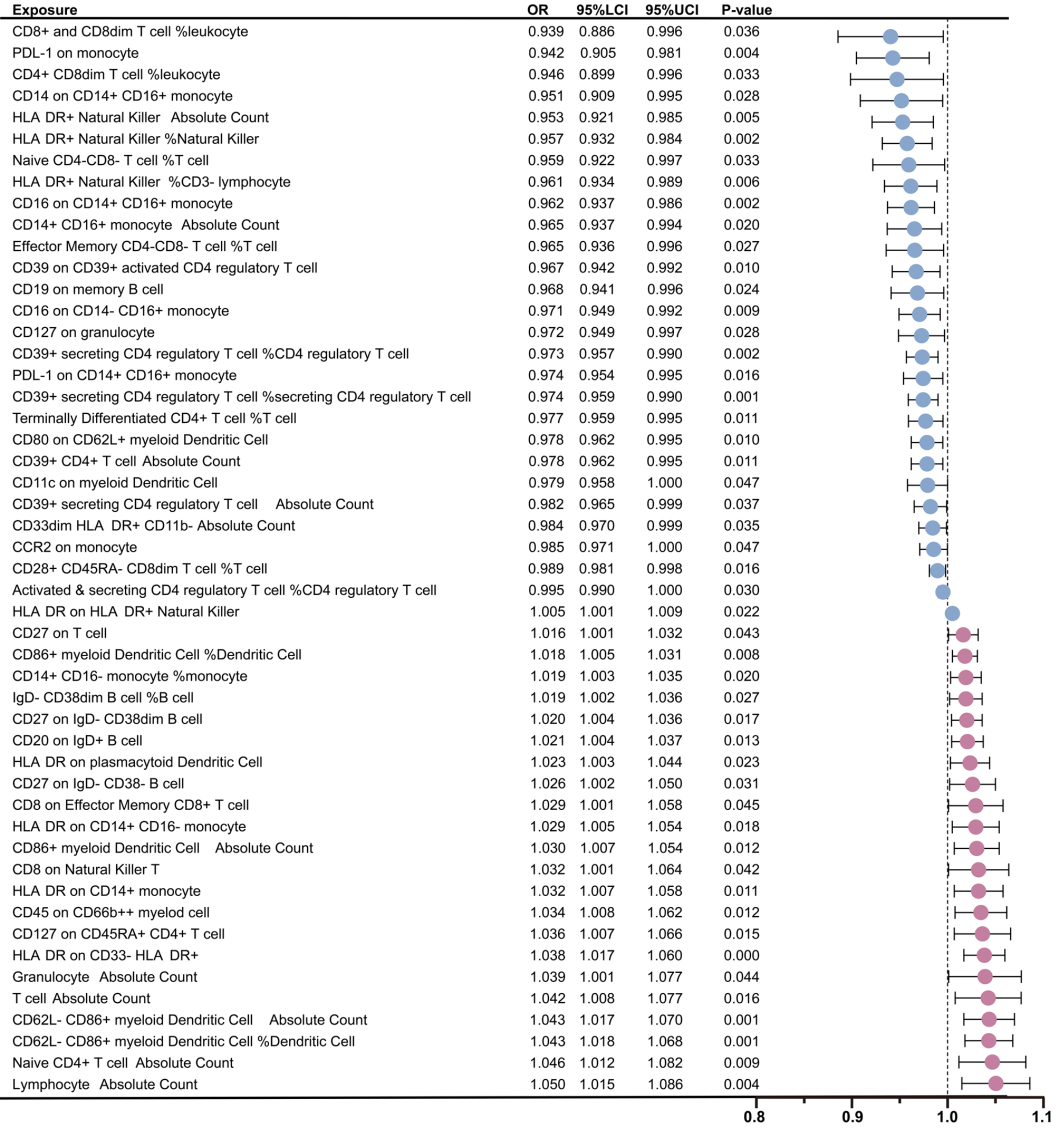
Mendelian randomization analyses show causal effects between immune cells and bacterial pneumonia. The dots colored in red and green indicate positive and negative ORs respectively from the IVW analysis (truncated at *P*-value < 0.05). OR, odds ratio; IVW, inverse-variance weighted; LCI, lower confidence intervals; UCI, upper confidence intervals

Following these GM findings, the relationship between immune cells and BP was rigorously examined using the IVW method. The analysis identified a total of 50 immune cell types significantly associated with BP, including 5 types within the B cell group, 18 within the TBNK group, 6 within the Treg group, 10 within the monocyte group, and 11 within the myeloid cell group (Supplementary file 1: Table S7). Among these identified immune cells, 27 types were deemed to have a protective effect against BP. Furthermore, gene prediction analysis revealed 23 types immune cell traits that may elevate the risk of BP (Fig. 3). In addition, the outcomes of the leave-one-out analyses demonstrated that the exclusion of individual SNPs could not influence the causality estimation (Supplementary file 2: Fig. S2). In the reverse MR analysis, we did not find substantial evidences to support a causal relationship between BP and the identified 50 immune cell types in the forward MR analysis. Detailed results of the reverse MR analysis were referred to Supplementary file 1: Table S8.

### Effects of GM on immune cells and mediation analyses of potential cells

**Fig. 4:**
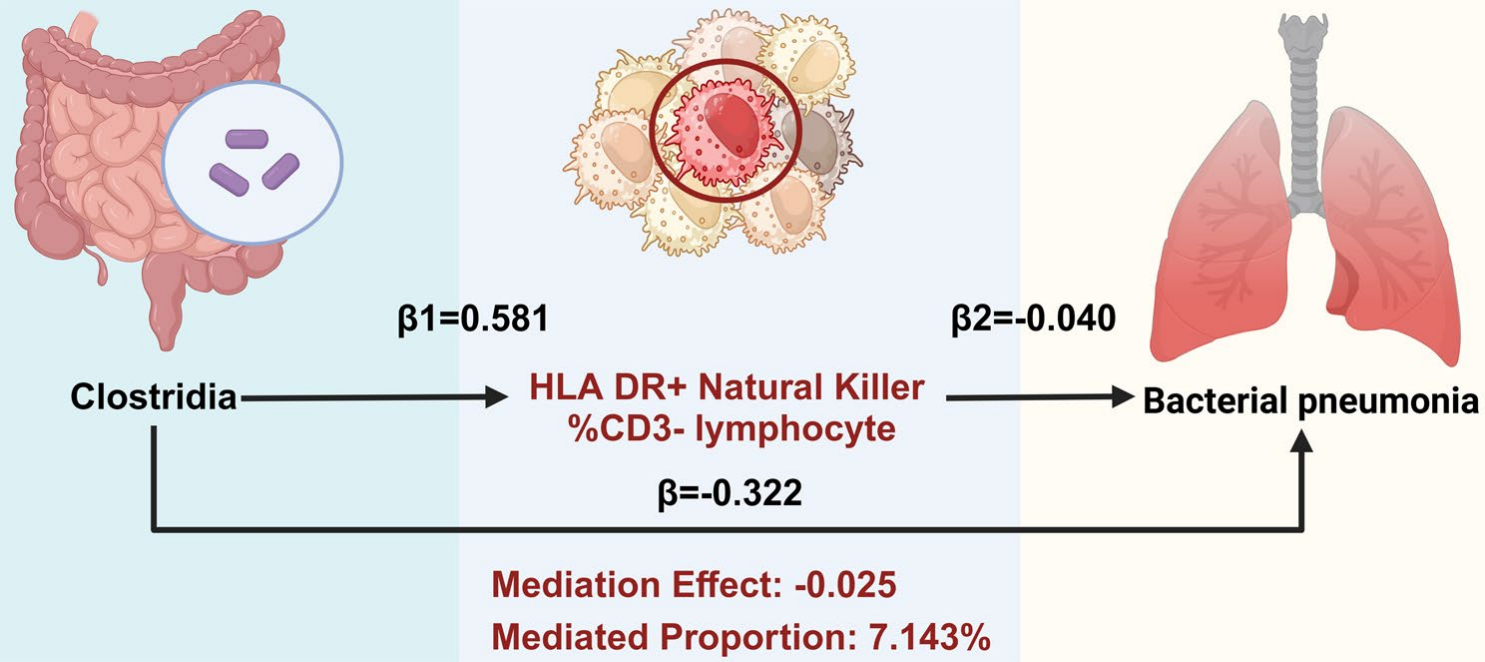
Mediation effect of Clostridia on bacterial pneumonia via the percentage of HLA DR + Natural Killer cells within CD3- lymphocytes

Building on these immune cell insights, 50 immunophenotypes were identified as potential mediators. Subsequently, MR analyses were conducted to examine the impact of the screened GM on these mediators. Utilizing IVW and MR Egger methods, we identified associations between 11 GM and 35 immune cell types (Supplementary file 1: Table S9). The results of the UVMR analysis indicated a significantly positive correlation between *Clostridia* microbiota and the percentage of HLA DR + Natural Killer cells within CD3- lymphocytes (HLA DR + Natural Killer %CD3- lymphocytes), with an OR of 1.788 (95% CI [1.012, 3.159], *P* = 0.045) (Supplementary file 1: Table S9). Furthermore, the MR analysis was conducted to assess the mediating role of HLA DR + Natural Killer %CD3- lymphocytes in the relationship between GM and BP. The evaluation of the mediation effect revealed an effect size of -0.025 (95% CI [-0.061, -0.000], with a mediation effect ratio of 7.143% (Fig. 4). The findings suggest that *Clostridia* confers a protective effect against BP via HLA DR + Natural Killer cells within CD3- lymphocytes.

### MR sensitivity analysis

To further validate these results, in the MR analysis of GM and BP, Cochran’s Q statistic, the MR-Egger intercept test, and MR-PRESSO identified one IV exhibiting heterogeneity and one IV demonstrating horizontal pleiotropy. All other *P*-values exceeded 0.05, indicating no significant heterogeneity or horizontal pleiotropy (Supplementary file 1: Table S10-S12). Similarly, in the MR analysis of immune cells and BP, one IV was found to be heterogeneous, while all other *P*-values were greater than 0.05. No significant heterogeneity or horizontal pleiotropy was observed (Supplementary file 1: Table S13-S15). In the MR analysis of GM and immune cells, Cochran’s Q statistic and the MR Egger intercept test identified certain IVs exhibiting potential heterogeneity and horizontal pleiotropy. These IVs were subsequently excluded from the final analysis. The remaining *P*-values exceeded 0.05, indicating no significant heterogeneity or horizontal pleiotropy (Supplementary file 1: Table S16 and S17). Overall, sensitivity analysis corroborated the robustness of the MR findings.

## Discussion

BP is a pulmonary disease resulting from bacterial infection. The GM has the capacity to modulate the host immune system through several mechanisms, consequently influencing the progression of BP [37–40]. In this study, our analysis revealed that *Clostridia* exert a protective effect against BP, as indicated by an OR of 0.976 associated with HLA DR + Natural Killer %CD3- lymphocytes utilizing MVMR methods alongside mediated MR techniques. These finding suggested that *Clostridia* may bolster host immune responses to BP by modulating the activity of HLA DR + Natural Killer within CD3- lymphocytes, thereby enhancing the overall immune response to this infection.

The gut microbiota plays a critical role in regulating gastrointestinal tract functions and also influences respiratory health and disease, thereby establishing the “gut-lung axis” [41]. *Clostridia*, a group of Gram-positive anaerobic bacteria, are essential components of the GM [42]. Empirical studies suggest that these bacteria can modulate the host’s immune system through a range of complex biological mechanisms [43]. Importantly, *Clostridia* have been demonstrated to promote the differentiation of regulatory T cells (Tregs), which are vital for maintaining immune homeostasis [44]. Simultaneously, *Clostridia* mitigate excessive inflammatory responses by decreasing the production of inflammatory mediators [45]. Furthermore, short-chain fatty acids produced by *Clostridia*, such as butyric acid, have been demonstrated to positively influence immune cell regulation. These fatty acids can not only modulate immune cell activity but also enhance the integrity of the intestinal epithelial barrier, thereby reducing the risk of pathogen invasion [46]. Studies have demonstrated that short-chain fatty acids activate heterotrimeric G proteins—composed of Gαi and Gβγ subunits—via their G protein-coupled receptors, primarily Free Fatty Acid Receptor 2 (FFAR2). The Gβγ subunits subsequently trigger downstream signaling pathways, notably PLCβ and PI3K. Consistent with this mechanism, short-chain fatty acids enhance the activation of key signaling molecules, including ERK/ATF2, STAT3/STAT5, and mTOR. The coordinated activation of these pathways effectively promotes the proliferation of intestinal innate lymphoid cells (ILCs, such as Natural Killer cells), thereby reinforcing mucosal immune responses [47].

HLA-DR + Natural Killer cells within CD3- lymphocyte population are integral to the innate immune system, possessing the capability to eliminate pathogens and tumor cells [48]. HLA-DR molecules, classified as major histocompatibility complex (MHC) class II molecules, are predominantly expressed on dendritic cells (DCs) and macrophages, as well as other antigen-presenting cells. Notably, Natural Killer cells can also express HLA-DR molecules under specific conditions [49]. It has been proposed that HLA-DR + Natural Killer cells are pivotal in immune regulation, exerting significant influence on the adaptive immune response via cytokine release and interactions with other immune cells [50].

The identified interplay between *Clostridia* and HLA DR + Natural Killer %CD3- lymphocytes in BP underscores a complex bidirectional crosstalk between gut microbiota (GM) and host immunological regulation. Within a translational epidemiology framework, the two-step MR-derived mediation effect (β = -0.025) indicates each standard deviation (SD) increase in *Clostridia* abundance reduces BP risk by 0.025 probability units through HLA DR + Natural Killer cells within CD3- lymphocytes mediation, equivalent to preventing 25 cases per 100,000 person-years—comparable to the efficacy of pneumococcal vaccination in transplant recipients [51, 52]. The mediation proportion of 7.14% shows that this pathway accounts for 1 in 14 preventable cases, analogous to the proportion of influenza protection mediated by CD8^+^ T cells [53].While the confidence interval approximating zero (-0.061 to -0.000) necessitates cautious interpretation, the negative directional trend of the point estimate corresponds with established gut-immune axis mechanisms [54]. Future studies require larger cohorts and enhanced measurement precision to validate this mediation pathway. Concurrently, targeted modulation of gut-immune interactions could complement existing preventive modalities to optimize population health outcomes.

**Fig. 5:**
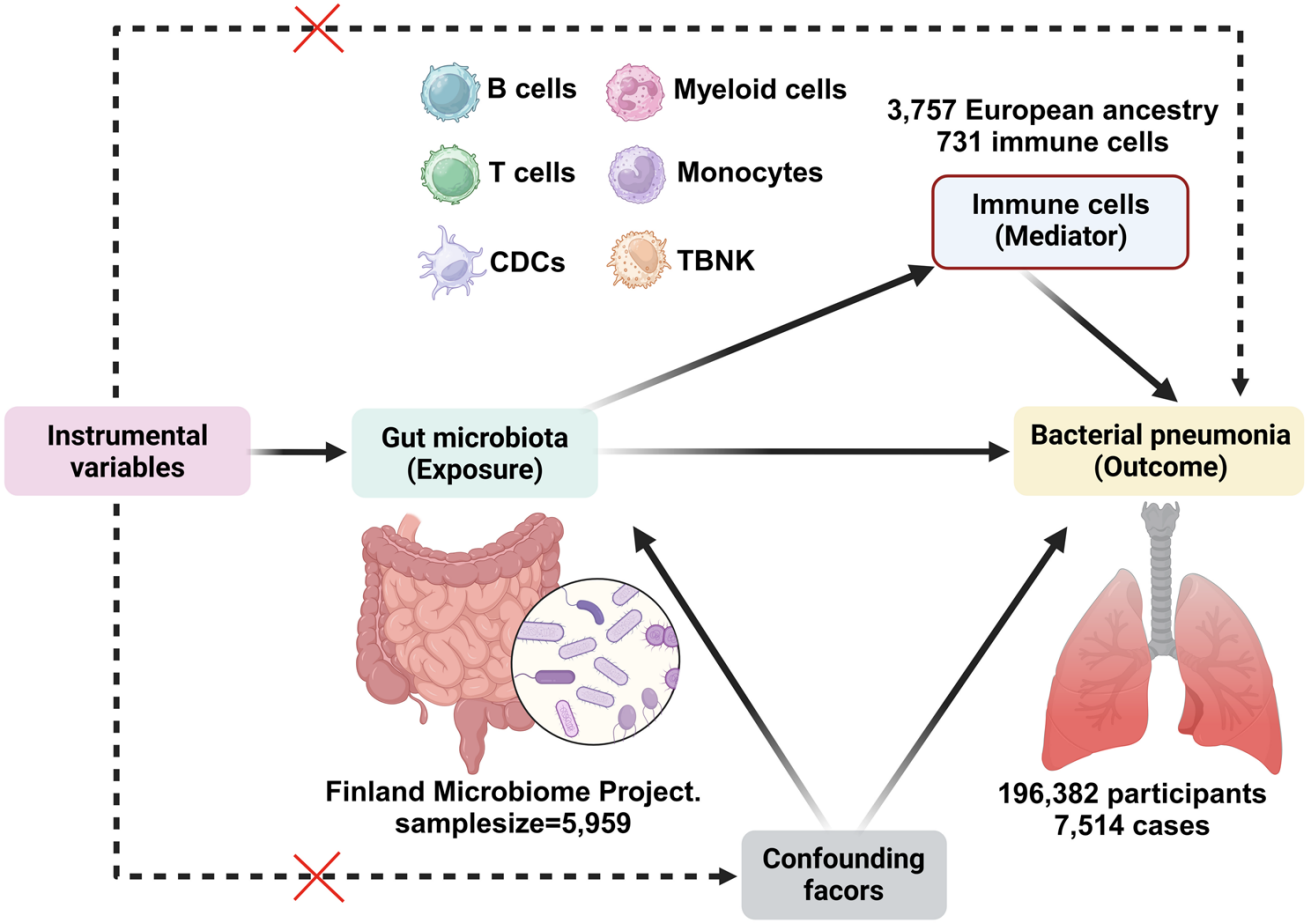
The study design. A two-step MR study of GM on BP mediated by immune cells

Existing observational data indicate that specific commensal microorganisms may attenuate the incidence of respiratory infections across bacterial and viral etiologies, whereas more robust clinical trial evidence demonstrates probiotic interventions effectively reduce both severity and duration of such infections in human cohorts [55, 56]. Experimental models have conclusively established the protective capacity of defined lactic acid bacteria and bifidobacteria strains against respiratory pathogen colonization and dissemination [57, 58]. The strengths of our study are manifold, with the design shown in Fig. 5. Firstly, it leverages a large-scale GWAS dataset that amalgamates pooled data from GM, immune cells, and BP analyses. This comprehensive approach not only yielded substantial findings but also provided robust statistical validation for the study’s outcomes. Secondly, our study presents a meticulously constructed analytical framework to investigate the potential causal relationship between GM and BP. By employing advanced methodologies such as UVMR and MVMR, our study elucidated the positive protective effect of *Clostridia* against BP, mediated via HLA DR + Natural Killer %CD3- lymphocytes. Finally, our study utilized a range of MR analysis techniques for causal inference and conducted sensitivity tests to assess the robustness of the findings. These methodological approaches ensured the reliability of the results by mitigating the influence of horizontal pleiotropy and other potential confounding factors.

Despite employing a genetically rigorous causal framework, our study is subject to interpretative caveats. The reliance on linear UVMR/MVMR models may inadequately represent nonlinear or environmentally contingent mechanisms underlying GM-BP interactions, as these approaches do not explicitly integrate gene-environment interplay or polygenic adaptation processes. Equally consequential is the restricted inclusion of European-ancestry cohorts, which limits extrapolation to global populations given ethnically stratified GM ecosystems (e.g., *Prevotella*-dominant enterotypes in Asian/African demographics [59]) and immunogenetic divergence (e.g., HLA-driven mucosal immunity [60])—critical determinants of microbiota-pathogen dynamics.

To address these limitations, future research should prioritize multi-ethnic GWAS and MR analyses that harmonize GM and disease phenotype data from diverse biobanks, distinguishing population-specific from conserved causal pathways in BP. External validation in non-European cohorts should refine confounder adjustment models to address ancestral heterogeneity. Culturally mediated modulators of GM-immune interactions—such as dietary practices (fermented food gradients) and probiotic use—require mechanistic dissection through integrated multi-omics approaches (metagenomics, metabolomics, single-cell immune profiling) to clarify their roles in pneumonia pathogenesis. Finally, genetically admixed populations could serve as natural experiments, leveraging Mendelian admixture mapping to disentangle genetic and environmental drivers of GM-pneumonia associations.

By operationalizing these strategies, researchers will enhance the generalizability of Mendelian randomization (MR)-based causal inferences while accelerating the development of ancestry-informed precision therapeutics. For instance, population-specific GM features could inform precision interventions, such as regionally adapted probiotic formulations or dietary modifications. Subsequent multi-center randomized trials should evaluate these interventions while accounting for host genetic and immunological diversity, ultimately establishing evidence-based strategies to mitigate the bacterial pneumonia burden.

## Conclusion

Our mediation analysis utilizing MR elucidated the causal relationship among GM, immune cells, and BP. Specifically, *Clostridia* demonstrated protective effects against BP through HLA DR + Natural Killer %CD3- lymphocytes, offering a novel perspective on the interplay between GM and the host immune system. Furthermore, this finding suggests a potential new avenue for the development of prevention and treatment strategies for BP.

## Electronic supplementary material

Below is the link to the electronic supplementary material.

Supplementary Material 1

Supplementary Material 2

## Data Availability

The dataset under analysis in the present study is accessible on the GWAS public website. The original contributions presented in the study are included in the article/Supplementary files.

## Abbreviations

BP: Bacterial pneumonia
GM: Gut microbiota
MR: Mendelian randomization
IVs: Instrumental variables
UVMR: Univariate Mendelian randomization
SNPs: Single nucleotide polymorphisms
MVMR: Multivariate Mendelian randomization
GWAS: Genome-wide association study
IVW: Inverse variance weighting
OR: Odds ratios
